# How resource sharing resists scarcity: the role of cognitive empathy and its neurobiological mechanisms

**DOI:** 10.1093/cercor/bhac017

**Published:** 2022-02-04

**Authors:** Fang Cui, Xiaoxuan Huang, Yiming Jing, Yue-jia Luo, Jie Liu, Ruolei Gu

**Affiliations:** School of Psychology, Shenzhen University, Shenzhen 518060, China; Center for Brain Disorders and Cognitive Neuroscience, Shenzhen University, Shenzhen 518061, China; School of Psychology, Shenzhen University, Shenzhen 518060, China; CAS Key Laboratory of Behavioral Science, Institute of Psychology, Chinese Academy of Sciences, Beijing 100101, China; Department of Psychology, University of Chinese Academy of Sciences, Beijing 100049, China; School of Psychology, Shenzhen University, Shenzhen 518060, China; Center for Brain Disorders and Cognitive Neuroscience, Shenzhen University, Shenzhen 518061, China; Center for Brain Disorders and Cognitive Neuroscience, Shenzhen University, Shenzhen 518061, China; School of Psychology, Shenzhen University, Shenzhen 518060, China; CAS Key Laboratory of Behavioral Science, Institute of Psychology, Chinese Academy of Sciences, Beijing 100101, China; Department of Psychology, University of Chinese Academy of Sciences, Beijing 100049, China

**Keywords:** resource scarcity, resource sharing, functional magnetic resonance imaging, empathy, oxytocin

## Abstract

Resource scarcity challenges individuals’ willingness to share limited resources with other people. Still, lots of field studies and laboratory experiments have shown that sharing behaviors do not disappear under scarcity. Rather, some individuals are willing to share their scarce resources with others in a similar way as when the resource is abundant, which is crucial for the maintenance and development of human society. Here, we designed a novel paradigm in which subjects decided whether (and how much) to share an amount of “relieving resources” for counteracting unpleasant noises, which mimics real-life situations that people cost their own resources to help others escape from adversity. Overall, the robustness of resource sharing under scarcity was positively correlated with individual level of the cognitive component of empathy across two independent experiments. Resource insufficiency modulated the activations of several brain regions (including the TPJ, mPFC, and PCC) as well as the functional connection (from the rTPJ to the mPFC) within the mentalizing brain network, but the modulatory effect decreased as a function of cognitive empathy. We also applied the administration of oxytocin and found significant effects on sharing behavior among individuals with a higher level of cognitive empathy, but not their low-level counterparts. These findings highlight the importance of empathy to resource sharing under scarcity and explain the underlying neurobiological mechanisms.

## Introduction

Human beings manifest a universal tendency of sharing resources with other people beyond blood ties (Aknin et al. 2013). Reciprocal or not, prosocial sharing happens in many ways such as gift-giving, lending, and charity donation (Harvey et al. 2020). The current study focuses on how resource scarcity influences sharing behavior, to what extent the robustness of sharing behavior depends on individual difference of empathy, and whether that robustness could be strengthened by endocrinological manipulation. Findings on these issues may enrich the knowledge about how to promote prosociality in real life (Xygalatas et al. 2013).

### Resource scarcity

As human ancestors underwent fluctuating ecological conditions, insufficiency of resources (food, medicine, territory, etc.) for survival and development has always been an important pressure source throughout history (Chakravarthy and Booth 2004; Laran and Salerno 2013). The experience of insufficient resources may create a “scarcity mindset,” that is shifting the attention away from other aspects to the scarce resource (Shah et al. 2015) and overvaluing that resource (Zhu and Ratner 2015; Hamilton et al. 2019). Thus when facing shortages, the willingness to share resources with other people significantly declines regardless of the importance of sharing behavior to social bonds (De Waal 1989; Briers et al. 2006).

Nevertheless, a large number of studies have shown that though prosocial sharing is suppressed under resource scarcity in general, but it is still reserved, or even strengthened sometimes, which is particularly true for some individuals (for a review, see Cannon et al. 2019). For instance, when subjects realized that the items they received were scarce, they were more likely to donate them back compared with the nonscarce condition (Louie and Rieta 2018). Various accounts have explained why and how resource sharing persists under shortage. A straightforward interpretation attributes this phenomenon to self-interest, that is people share resources in anticipation of reciprocation (Raihani and Bshary 2015; Sebastián-Enesco and Warneken 2015). However, it is not uncommon to share resources with unrelated individuals unconditionally even though people are suffering from scarcity (Smith and Bird 2000; Lotem et al. 2003; Haidt 2007). In this regard, the importance of social emotions especially empathy should be highlighted.

### Empathy and oxytocin

Empathy is not a unitary concept; instead, it comprises multiple components (Singer 2006; Decety and Meyer 2008; Decety and Cowell 2014). On one hand, the affective dimension of empathy refers to the ability to emotionally “resonate” with other people’s feelings, including the experience of sharing other persons’ internal states (Zaki and Ochsner 2012). On the other hand, the cognitive dimension of empathy refers to the ability to perceive and know others’ cognitive mental states (e.g. intentions, needs, and beliefs), including mentalizing and perspective-taking (Shamay-Tsoory et al. 2009; Shamay-Tsoory 2011; Perez-Manrique and Gomila 2018). The neural circuits implicated in empathy consist of a wide range of brain regions associated with these two components (Singer 2006; Decety 2011; Walter 2012; Zaki and Ochsner 2012). The medial cingulate cortex and limbic regions (e.g. amygdala, anterior insula) are suggested to be related to the affective component (Decety 2011; Lamm et al. 2011; Walter 2012). Meanwhile, the temporoparietal junction (TPJ), medial prefrontal cortex (mPFC), and posterior cingulate cortex (PCC) are more likely to be associated with the cognitive aspect that is responsible for mentalizing and social attributions (Molenberghs et al. 2016; Bellucci et al. 2020). The TPJ has been frequently reported in social decision-making tasks especially when those tasks engage other-regarding considerations (e.g. charity: see Morishima et al. 2012; Tusche et al. 2016; Park et al. 2017). The mPFC has been suggested to be engaged in belief-desire reasoning (Frith and Frith 2001, 2006), as well as in computing “social values” during social interactions (Behrens et al. 2008). The PCC has been reported to be involved in motor imagery and judging other’s visual perspective, and thus, its main function may be associated with the mental representation of others during metalizing (Suchan et al. 2002; Hanakawa et al. 2003; Cavanna and Trimble 2006).

Empathy is considered to be a hallmark of psychological maturity since it allows people to understand how others are affected by an event, which then facilitates social interaction and creates social coherence (de Vignemont and Singer 2006). Empathic processes give rise to prosocial behaviors including altruistic sharing, helping, and caring (Preston and de Waal 2002; De Waal 2008; FeldmanHall et al. 2015; Zaki 2020). Seeing that empathy generates a pure other-oriented motive to help others (Eisenberg 2000), we suggest that empathy has accounted for the inconsistent findings of sharing behavior under resource scarcity in previous studies (see above). That is to say, people with a higher level of empathy trait should be more likely to share scarce resources with other people.

Empathy itself, however, is a limited resource and therefore may not be sufficient even when others are suffering (Decety and Cowell 2014). Here, we applied intranasal oxytocin administration as a means to enhance empathic responding. Oxytocin has been related to various forms of sociality, including trust, generosity, and other-regarding preferences (De Dreu et al. 2010; Quintana et al. 2015; Ma et al. 2016; Wang and Ma 2020). Also, oxytocin is considered to be a mediator of empathy, as it underlies the behavioral states and responses necessary for empathy (Barraza and Zak 2009; Shamay-Tsoory et al. 2013; Smith et al. 2014; De Dreu and Kret 2016).

### Experimental hypotheses

Various kinds of experimental manipulation of scarcity (e.g. triggering thoughts about the past when resources were scarce) have been applied in previous studies (for a review, see Cannon et al. 2019). However, Huijsmans et al. (2019) pointed out that these manipulations: (i) do not always ask subjects to decide about actual resources at hand and (ii) might be confounded by subjects’ different life histories. More importantly, sharing money, which has been used in the experimental designs of most previous studies, may have limited implication about how people would share resources to against physical threat in real life (e.g. sharing food during a famine, sharing medicines during a pandemic).

For these concerns, we designed a novel paradigm in which a pair of players was exposed to the threat of receiving a certain duration of highly unpleasant noise; one of the players (i.e. the subject) would decide whether to help her/his partner reducing noise duration by sharing some “relieving resources” that were only endowed to her/himself. We manipulated the amount of resources being endowed to generate abundant or scarce situations. This task shares key elements with a variant of the ultimatum game that has been used to investigate social norm compliance (Spitzer et al. 2007; Ruff et al. 2013). In experiment 1, we combined our paradigm with functional magnetic resonance imaging (fMRI) to investigate sharing decisions under resource scarcity, its sensitivity to empathy, and the associated brain mechanisms. Then, in experiment 2, we observed the effect of oxytocin on sharing resources under different levels of empathy trait. We have three hypotheses: (i) on the behavioral level, resource sharing would be suppressed under scarcity compared with abundance. Furthermore, we predicted that the influence of resource scarcity on sharing behavior would vary as a function of individual level of empathy trait. (ii) On the neural level, we expected that resource scarcity would show a modulating effect on the activation level of the brain regions associated with empathy (i.e. the TPJ, mPFC, and PCC), as well as the functional connectivity between these regions. (iii) Finally, we predicted that oxytocin administration would promote sharing behavior, even under resource scarcity. Specifically, our experiment 1 aimed to examine hypotheses (1) and (2), while experiment 2 mainly focused on hypothesis (3).

## Experiment 1

### Methods

#### Subjects

A priori power analysis conducted using the G^*^Power 3.1 revealed that 23 subjects were required to reach a good statistical power of 0.9 to detect median-sized (*f* = 0.25) effects with an alpha value of 0.05 for a one-factor within-subjects analysis of variance (ANOVA). To account for possible dropouts or errors during the experiment, 35 right-handed subjects were recruited from Shenzhen University to join in the fMRI experiment. Subjects were screened for a history of neurological disorders, brain injury, and developmental disabilities. All had normal or corrected-to-normal vision. Three of them who had excessive head movements >2°in rotation or >2 mm in translation during the scanning were excluded, leaving 32 subjects in the final sample (15 women, age: 20.10 ± 1.34 years). The study was conducted according to the ethical guidelines and principles of the Declaration of Helsinki and was approved by the Medical Ethical Committee of Shenzhen University Medical School. Informed consent was obtained from all subjects after they fully understood the procedures.

#### Experimental design

Upon arrival at the laboratory, each subject was introduced to another player (who was a confederate of the same sex). The two of them drew lots to decide their roles in the formal task, which were manipulated such that the real subject always played as a “resource owner.” During the whole task, the real subject would be in the MRI scanner and s/he was informed that the confederate was seated in another room.

Before the formal experiment started, both players heard 30 one-second noise clips of varying loudness in a randomized order and rated the unpleasantness of each clip on an 11-point visual analog scale from 0 (not unpleasant at all) to 10 (extremely unpleasant) (Hu et al. 2017, 2021). The noise stimuli were delivered by AKG K271 MKII headphones and controlled by E-prime 2.0 (Psychology Software Tools, Inc., Pittsburgh, PA). Noise stimuli would then be tailored to the results of the emotional rating, such that the noise administration for each subject during the formal task (see below) was equivalent to the noise clip rated as level 8 (i.e. highly unpleasant) by that specific subject.

As described in Introduction section, this newly designed paradigm simulates a social dilemma that resources are insufficient for two persons to deal with a potential threat. Before the task, the real subject was told that both her/him and the confederate would receive a short duration of unpleasant noise administration, but prior to that s/he (as resource owner) would be endowed with a certain amount of relieving resource (i.e. a few seconds to spare the noise), whereas the confederate received none. In each trial of the task, the real subject should decide how many resources s/he would like to keep for her/himself, and the rest would be given to the other player automatically. According to the real subject’s knowledge, her/his decision would be confidential to that player. Only one randomly selected trial would be executed after the task. Still, the best strategy for the subject was to treat each trial equally (Knutson et al. 2007).

Both the real subject (resource owner) and the confederate received 8 s of unpleasant noise in each trial; then, the resource owner was endowed with a certain amount of “relieving resource” (16, 8, or 4 s), while the confederate received none. The resource owner could decide how many seconds of reliving resources s/he want to share. The experiment applied a one-factor (“resource sufficiency” sufficient: 16 s; insufficient: 8 s; highly insufficient: 4 s) within-subject design. Please note that “sufficiency” here was defined according to whether the relieving resources were enough for both players.

**Fig. 1:**
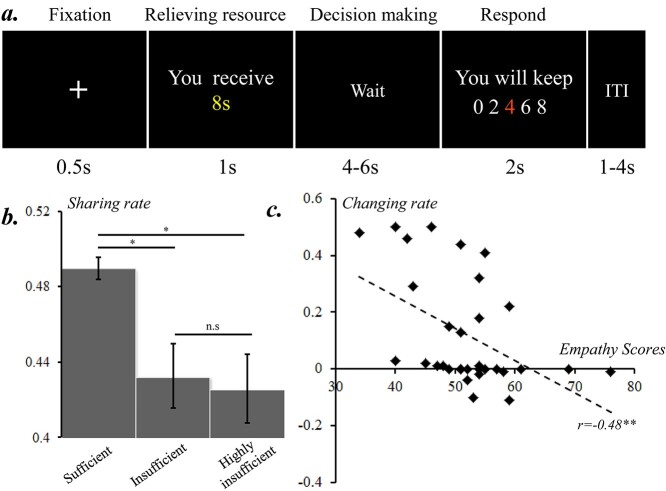
Experimental design and behavioral results of experiment 1. a) Structure of an example trial; b) results of the sharing rate for each level of resource sufficiency; c) correlations between the changing rate and cognitive component of empathy score; n.s.: not significant. ^^*^^: *P* < 0.05. ^^*^^*^^: *P* < 0.01.

In each trial, after a 0.5-s fixation, each subject observed how many seconds of relieving resource s/he was endowed in this trial (16, 8, or 4 s), the presentation of which lasted for 1 s. After a 4- to 6-s wait, during which subjects could make their decisions, five options ranging from 0 to 100% with a step of 25% were horizontally presented. Note that the five options were converted to seconds, not percentages (Fig. 1a). The order of the five options was either monotonically increased or decreased (randomized trial by trial). The color of the chosen option would turn red from white. To avoid any priming effect, the starting position of the red label (indicating the chosen option at the time) was pseudo-randomized (see also Liu et al. 2020) such that it randomly appeared on one of five positions with an equal probability (i.e. 20%). The subject first moved the red label to the left or right side by pressing one of two pre-assigned buttons on an MRI-compatible button-box, then pressed the third button to confirm her/his choice. The subject had 2 s to finish the response; otherwise, no resource would be kept for her/him. The intertrial interval (ITI) was 1–4 s. A pilot behavioral experiment with 40 right-handed subjects has verified the validity of the experiment settings (see Supplementary 1).

The experiment consisted of two runs of 45 trials lasting for ~30 min. Each run included 15 trials of each condition, the sequence of which was pseudo-randomized. Thus, each condition contained 30 trials in total. Before the scanning, each subject was familiarized with the task with a practice block consisting of eight trials. After the experiment, the subjects received remuneration and a debriefing was given by the experimenter. Additionally, to access whether individual empathic traits play a role in solving this resource dilemma, all the subjects were asked to fulfill the Questionnaire of Cognitive and Affective Empathy (QCAE; Reniers et al. 2011) during the recruiting process. The same was true for the follow-up experiment 2.

#### Neuroimaging data acquisition and preprocessing

We used a Siemens TrioTim 3.0 T MRI machine for data acquisition. Functional volumes were acquired using multiple slice *T*_2_-weighted echo planar imaging sequences with the following parameters: repetition time = 2000 ms, echo time = 30 ms, flip angle = 90°, field of view = 224 × 224 mm^2^, 33 slices covering the entire brain, slice thickness = 3.5 mm, and voxel size = 3.5 × 3.5 × 3.5 mm^3^.

fMRI data were preprocessed in SPM12 (Wellcome Department of Imaging Neurosciences, University College London, UK, http://www.fil.ion.ucl.ac.uk/spm). Images were slice-time corrected, motion-corrected, and normalized to the Montreal Neurological Institute (MNI) space for each subject with a spatial resolution of 3 × 3 × 3 mm^3^. Images were then smoothed using an isotropic 6-mm Gaussian kernel and high-pass filtered at a cutoff of 128 s.

#### Statistical analysis

##### General linear model

Statistical parametric maps were generated on a voxel-by-voxel basis with a hemodynamic model to estimate brain response. The time period of fixation, the endowed resource, the order of options, and the ITI were included in the general linear model (GLM) at the single-subject level. The six rigid body parameters were also included in the GLM to exclude head-motion nuisance. The effect of the experimental conditions on regional blood oxygenation level-dependent responses was estimated with the GLM using the three levels of relieving resource (sufficient, insufficient, highly insufficient) as independent regressors. Further analysis of brain activation was based on these three regressors. Single-subject *t*-contrasts were computed for the three conditions. Our main interest focused on the contrast between sufficiency and insufficiency (including the “insufficient” and “highly insufficient” conditions), and thus, we defined two contrasts: (2 × sufficient condition − [insufficient condition *+* highly insufficient condition]) and ([insufficient condition *+* highly insufficient condition] − 2 × sufficient condition). We also compared brain activation between either two of the three conditions (i.e. sufficient, insufficient, and highly insufficient) (for details see Supplementary 2). For the group-level analysis, a one-sample *t*-test was conducted using the whole brain as the volume of interest. The significance level was set to *P* < 0.001 uncorrected at the voxel level and to an extent threshold of *P* < 0.05 with family-wise error (FWE) correction at the cluster level in the univariate analyses.

##### Effective connectivity analysis: dynamic causal modeling

We used dynamic causal modeling (DCM; Friston et al. 2003) and hypothesized that the modulation effect on effective connectivity between brain regions sensitive to resource sufficiency [i.e. the right TPJ (rTPJ) and mPFC; see below] would show different patterns when comparing between the sufficient condition with the insufficient/highly insufficient conditions. According to our results, the rTPJ and mPFC were consistently activated in all of the three contrasts, that is (2 × sufficient condition − [insufficient condition + highly insufficient condition]), (sufficient condition − insufficient condition), and (sufficient condition − highly insufficient condition); in contrast, the PCC was not significantly activated in the contrast of (sufficient condition − insufficient condition). These results suggest that unlike the rTPJ and mPFC, the PCC was not consistently sensitive to the difference between sufficiency and insufficiency. Therefore, we decided to use a more parsimonious DCM model including the rTPJ and mPFC but not the PCC.

We used DCM12 to examine the effective connectivity between the rTPJ and mPFC during the task (Friston et al. 2003). The first eigenvariate for the single-subject time courses was extracted from volumes located in the rTPJ and mPFC. To ensure that the functional regions were consistent across subjects, region of interest (ROI) selection was guided by group results of the contrast (2 × sufficient condition − [insufficient condition + highly insufficient condition]). Subject-specific ROIs (6-mm sphere) were defined as the local maxima of these two regions with a liberal threshold of *P* = 0.05 uncorrected. We extracted time series from each subject’s activation map at the closest maxima within a distance of 6 mm of the group peak voxel. The ROI time series were extracted from the whole-brain activation in the sufficient, insufficient, and highly insufficient conditions.

To determine the driving input (matrix C) and modulatory effect (matrix B), we fixed the effective connectivity between the two regions (i.e. the rTPJ and mPFC) as bilateral connections. Our model space consisted of 9 models in total, which were differentiated by where the modulatory effect (matrix B) took place and where the input went into (each node, respectively, or both nodes). We included all possible unidirectional/bidirectional modulations between the rTPJ and the mPFC (see Supplementary 3). All the 9 models were specified separately for each run and each subject. We then estimated all the models and subjected them to random-effect Bayesian model selection (BMS) to select the best-fitted model from our model space based on the model evidence (Stephan et al. 2010). Bayesian model averaging (BMA) was then used to calculate weighted-model parameters for the winning model.

## Results

### Behavioral results

On average, the subjects did not respond in 1.15 ± 1.80 trials (mean ± S.D.; range: 0–7), and 20 out of 35 subjects did not miss any trial, and thus, there were sufficient trials in each condition for data analysis.

Two behavioral indexes were used to describe the sharing behavior, that is sharing rate and changing rate. The sharing rate was calculated as “the amount of resource each subject shares with the other player” divided by “the total amount of resource” in any given trial. For example, if a subject received 8 s of relieving time, and s/he kept 6 s for her/himself (in other words, giving 2 s to the other player), and then, the sharing rate was 2/8 = 0.25 (25%). The sharing rate indicated subjects’ prosocial tendency when making sharing decisions. Meanwhile, the changing rate was calculated as: (2 × sharing rate_sufficient_ − [sharing rate_insufficient_ + sharing rate_highly insufficient_]), indicating the behavioral difference between sufficiency and insufficiency. A higher changing rate indicates a stronger modulating effect of resource scarcity on sharing decision in general.

Repeated measures ANOVA was conducted on the sharing rate under different levels of resource sufficiency. The main effect of resource sufficiency was significant (*F*(2, 62) = 10.84, *P* < 0.001, *η_p_^2^* = 0.26); the sharing rate was higher when resource was sufficient than when it was insufficient (sufficient: 0.49 ± 0.01, insufficient: 0.43 ± 0.02, highly insufficient: 0.43 ± 0.02; sufficient vs. insufficient: *P* = 0.002; sufficient vs. highly insufficient: *P* = 0.001; insufficient vs. highly insufficient: *P* = 0.40) (Fig. 1b). Pearson correlation analysis showed that the changing rate was negatively correlated with the scores of cognitive empathy measured with QCAE (*r* = −0.48, *P* = 0.005) (Fig. 1c).

### Univariate analysis

**Fig. 2:**
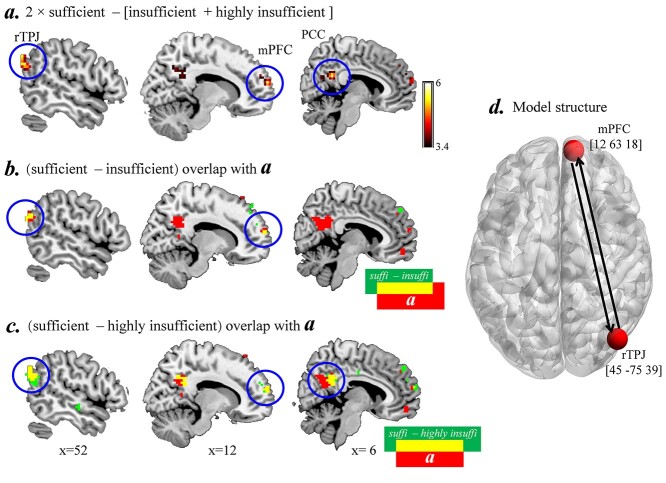
Brain-imaging results of experiment 1. a) Brain activation of (2 × sufficient condition − [insufficient condition *+* highly insufficient condition]); b) brain activation of (sufficient condition − insufficient condition) overlapped with the contrast of (2 × sufficient condition − [insufficient condition + highly insufficient condition]); c) brain activation of (sufficient condition − highly insufficient condition) overlapped with the contrast of (2 × sufficient condition − [insufficient condition + highly insufficient condition]); d) visual representation of the connections between nodes for DCM analysis.

The contrast of (2 × sufficient condition − [insufficient condition *+* highly insufficient condition]) revealed significant activations in the mPFC (peak MNI [12 63 18], cluster size = 44, *t*(31) = 5.63), rTPJ (peak MNI [45 −75 39], cluster size = 63, *t*(31) = 5.47), and PCC (peak MNI [6 −45 27], cluster size = 83, *t*(31) = 5.22). The reversed contrast revealed significant activations in the left (peak MNI [−42 −75 −9], cluster size = 129, *t*(31) = 6.75) and right inferior occipital gyrus (IOG; peak MNI[48 −75 −3], cluster size = 77, *t*(31) = 6.37) (Fig. 2a; Table 1). Then, we compared brain activation between either two of the three conditions (i.e. sufficient, insufficient, and highly insufficient). The results showed that the contrasts of (sufficient condition − insufficient condition) and (sufficient condition − highly insufficient condition) yielded a similar pattern, that is stronger activations in the right TPJ and mPFC, but the PCC was only significant in the contrast of (sufficient condition − highly insufficient condition). The contrast of (insufficient condition − highly insufficient condition) revealed no significant difference (Fig. 2b and c, also see Supplementary 2 for details).

**Table 1:** Whole-brain activations based on the contrasts between the sufficient condition and the insufficient conditions.

Brain region	BA	Coordinates			Vol.	*T*-value
		(X, Y, Z)				
***2 × sufficient condition > insufficient condition + highly insufficient condition***						
mPFC^^*^^	10	12	63	18	44	5.63
	10	18	63	9		4.34
Right tempoparietal junction^^*^^	39	45	−75	39	63	5.47
	39	51	−69	30		4.87
PCC^^*^^	26	6	−45	27	83	5.22
		3	−54	30		3.95
		12	−57	36		3.88
Medial superior frontal gyrus	9	6	48	42	14	4.39
Left inferior temporal gyrus	20	−60	−18	−24	11	4.37
Medial superior frontal gyrus	32	9	54	27	10	4.19
Left tempoparietal junction	39	−51	−66	39		3.61
***2 × sufficient condition < insufficient condition + highly insufficient condition***						
Left inferior occipital gyrus^^*^^	19	−42	−75	−9	129	6.75
	19	−45	−72	0		
	37	−42	−60	−18		
Right inferior occipital gyrus^^*^^	19	48	−75	−3	77	6.37
	19	36	−81	−12		

*Note:* All the results reported above were significant at *P* < 0.001, uncorrected at the voxel level; the threshold of the cluster size was set as ≥10 voxels.

^^*^^Indicates the cluster-level FWE correction at *P* < 0.05.

We also run Pearson correlation tests between the brain activations extracted from three ROIs (the mPFC, rTPJ, and PCC) and cognitive empathy scores. For the mPFC, the activation level in all the three conditions showed significant correlations (uncorrected) with the empathy score (sufficient: *r* = 0.385, *P* = 0.030; insufficient: *r* = 0.390, *P* = 0.027; highly insufficient: *r* = 0.427, *P* = 0.015). For the rTPJ, a similar trend was observed but did not reach significance (sufficient: *r* = 0.301, *P* = 0.094; insufficient: *r* = 0.247, *P* = 0.127; highly insufficient: *r* = 0.330, *P* = 0.065). The same was true for the PCC (sufficient: *r* = 0.320, *P* = 0.074; insufficient: *r* = 0.273, *P* = 0.135; highly insufficient: *r* = 0.304, *P* = 0.102). None of these correlations survived multiple comparison correction.

### DCM results

We chose the significantly activated brain regions within all of the three contrasts between sufficiency and insufficiency as nodes in the DCM analysis, including the rTPJ [45 −75 39] and mPFC [12, 63, 18] (Fig. 2d). Two subjects were excluded as they did not show significant activation at the threshold of *P* = 0.05 in the two ROIs, and thus, the sample for this analysis included 30 subjects.

**Fig. 3:**
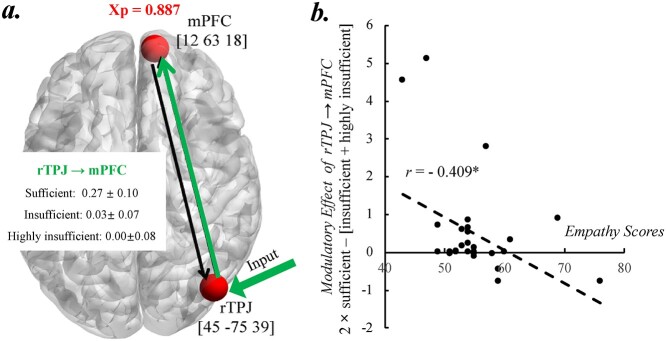
Results of DCM of experiment 1. a) Model structures of the best model (green solid line with arrow indicates where the modulatory effect tool place; green arrow indicates the input; xp: exceedance probability); b) correlations between the difference between sufficient and insufficient conditions in modulation strength of the rTPJ → mPFC and cognitive component of empathy scores.

The BMS result showed that the model No. 5 (i.e. the input went into the rTPJ and the modulatory effect on the connection from the rTPJ to the mPFC) offered the best fit to the data (exceedance probability [xp] = 0.887; Fig. 3a and Supplementary 3).

BMA was then used to calculate weighted-model parameters within the winning model for statistical analysis. First, we found that the modulatory effect on the connections from the rTPJ to the mPFC became weaker as a function of resource insufficiency. Specifically, in the sufficient condition, the modulatory effects were significantly larger than zero (0.265 ± 0.10, *t*(29) = 2.69, *P* = 0.011); while in the insufficient and highly insufficient conditions, the effects were no different from zero (insufficient: 0.03 ± 0.07, *t*(29) = 0.45, *P* = 0.65; insufficient: 0.00 ± 0.08, *t*(29) = 0.04, *P* = 0.97). Paired *t*-tests revealed that the modulatory effect in the sufficient condition was significantly stronger than that in the insufficient and highly insufficient conditions (sufficient vs. insufficient: *t*(29) = 2.28, *P* = 0.037; sufficient vs. highly insufficient: *t*(29) = 2.65, *P* = 0.016; insufficient vs. highly insufficient: *t*(29) = 0.68, *P* = 0.509) (Fig. 3a).

To further unravel how the strength of the modulatory effect varied according to the individual level of empathy trait, we calculated the correlations between the difference in the strength of modulatory effect between sufficient and insufficient conditions (i.e. 2 × sufficient condition − [insufficient condition + highly insufficient condition]) and empathy score. We found significant negative correlations between the empathy score (the cognitive component) and the difference in the strength of modulatory effect of the connection from the rTPJ to the mPFC (*r* = −0.409, *P* = 0.024) (Fig. 3b).

## Experiment 2

### Methods

#### Subjects

A priori power analysis conducted using the G^*^Power 3.1 revealed that 70 subjects were required to reach a good statistical power of 0.9 to detect median-sized (*f* = 0.25) effects with an alpha value of 0.05 for a 2 × 3 mixed ANOVA. Eighty right-handed subjects were recruited from local universities for participation in this oxytocin experiment. We recruited only male subjects to avoid potential confounds of sex differences in oxytocin effects (Liu et al. 2019; Zhu et al. 2020). None of these persons had participated in the pilot study or experiment 1. Three subjects were excluded due to the interruption of personal phone calls, leaving 77 subjects in the final sample (21.13 ± 1.93 years).

#### Experimental design

Subjects were randomly assigned to the intranasal administration of oxytocin or placebo in a double-blind placebo-controlled mixed design. All subjects were instructed to abstain from cigarettes, alcohol, and caffeine for 24 h before the experiment and to refrain from eating or drinking anything except water for 2 h before the experiment. Subjects self-administered one puff (i.e. 4 IU) of IN-OT (or placebo) every 30 s, alternating between nostrils; each individual received 40 IU of IN-OT (Syntocinon; Novartis, Basel, Switzerland) or placebo (same composition as Syntocinon except for OT) in total. The administration phase lasted ~9 min including a 3-min rest at the end; 35 min after receiving the dose, they were instructed to start the main task (Paloyelis et al. 2016; Liu et al. 2019). The main task was the same as that in experiment 1 except that the trial number for each condition was reduced to 10, leaving 30 trials in total to shorten the experiment.

## Results

For the sharing rate, repeated-measures ANOVA with “resource sufficiency” (sufficient, insufficient, highly insufficient) as a within-subject factor and “hormone treatment” (oxytocin vs. placebo) as a between-subject factor was conducted. Consistent with previous results, we found a significant main effect of resource sufficiency (*F*(2,150) = 27.57, *P* < 0.001, *η_p_^2^* = 0.72) such that subjects shared less resources with the other player when the resources were insufficient or highly insufficient (sufficient: 0.50 ± 0.00, insufficient: 0.39 ± 0.02, highly insufficient: 0.37 ± 0.02; sufficient vs. insufficient: *P* < 0.001; sufficient vs. highly insufficient: *P* < 0.001; insufficient vs. highly insufficient: *P* = 0.08). The main effect of hormone treatment or its interaction with resource sufficiency was not significant (*P*s > 0.49).

**Fig. 4:**
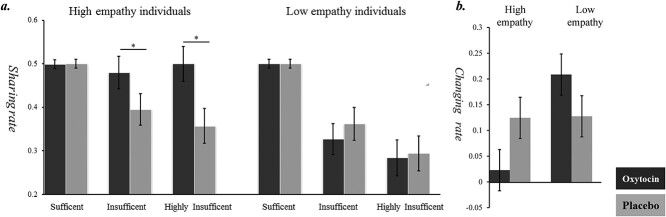
Results of experiment 2. a) Effects of oxytocin and cognitive empathy score on the sharing rate; b) effects of oxytocin and cognitive empathy score on the changing rate. Each box indicates the mean value of each group. n.s.: not significant. ^^*^^: *P* < 0.05.

Seeing that empathy, especially its cognitive component, played a prominent role in the relationship between resource sufficiency and sharing decision (see the results of experiment 1), we separated the subjects into a high- and a low-empathy group based on their score in the cognitive aspect of QACE. The low-empathy group included 38 subjects (20 subjects received oxytocin; empathy scores: 50.21 ± 4.09; range: 38–54), while the high empathy group included 39 subjects (19 subjects received oxytocin; empathy scores: 59.58 ± 4.42; range: 55–75). Then, we run a three-way ANOVA with resource sufficiency (3 levels: sufficient, insufficient, highly insufficient), hormone treatment (2 levels: oxytocin vs. placebo), and cognitive empathy trait (2 levels: high and low) on the sharing rate. Results showed that the main effect of resource sufficiency was significant (*F*(2,146) = 29.48, *P* < 0.001, *η_p_^2^* = 0.29); the main effect of cognitive empathy trait was also significant (*F*(1,73) = 6.39, *P* = 0.014, *η_p_^2^* = 0.08); the main effect of hormone treatment was not significant (*P* = 0.45). The two-way interactions resource sufficiency × cognitive empathy trait (*F*(2,146) = 6.05, *P* = 0.006, *η_p_^2^* = 0.08) and hormone treatment × cognitive empathy trait were both significant (*F*(1,73) = 5.14, *P* = 0.026, *η_p_^2^* = 0.07). The three-way interaction resource sufficiency × hormone treatment × cognitive empathy trait was also significant (*F*(2,146) = 4.83, *P* = 0.015, *η_p_^2^* = 0.06). Pairwise comparison on the three-way interaction revealed that for the high-empathy trait individuals, oxytocin significantly increased their sharing rate in the insufficient and highly insufficient conditions (highly insufficient: 0.50 ± 0.04 vs. 0.36 ± 0.4, *P* = 0.018, insufficient: 0.48 ± 0.04 vs. 0.40 ± 0.04, *P* = 0.047) but not in the sufficient condition (*P* = 0.94); meanwhile, for the low-empathy trait individuals, oxytocin showed no effect on the sharing rate in all the three conditions (*P*s > 0.13) (Fig. 4a).

Regarding the changing rate, we run a two-way ANOVA with hormone treatment (2 levels: oxytocin vs. placebo) and cognitive empathy trait (2 levels: high and low) as two between-subject factors. Results showed that the main effect of empathy trait was significant (*F*(1,73) = 7.98, *P* = 0.006, *η_p_^2^* = 0.10). The main effect of hormone treatment was not significant (*F*(1,73) = 0.31, *P* = 0.59, *η_p_^2^* = 0.08). The hormone treatment × cognitive empathy trait interaction was significant (*F*(1,73) = 6.05, *P* = 0.02, *η_p_^2^* = 0.08). Pairwise comparisons revealed that for the high-empathy trait individuals, oxytocin significantly reduced their changing rate (0.02 ± 0.04 vs. 0.12 ± 0.04, *P* = 0.035); for the low-empathy trait individuals, oxytocin showed no effect on the changing rate (0.21 ± 0.04 vs. 0.14 ± 0.04, *P* = 0.186) (Fig. 4b).

## Discussion

When resources are limited, it might be evolutionary adaptive to be sparing and self-protective. However, it is also important for community members to help each other going through challenges and disturbances; otherwise, the whole society may collapse (Smith et al. 1983). Therefore, altruistic sharing is valued as a traditional virtue across many cultures (Henrich et al. 2006) and it is theoretically meaningful to investigate its tenacity. In this study, we systematically explored the impact of resource scarcity on sharing behavior. Behavioral results of three experiments unanimously showed that compared with the sufficient condition, the sharing rate was lower when the resource was either insufficient or highly insufficient, possibly due to a scarcity mindset (Shah et al. 2012). According to Cannon et al. (2019), retaining more resources could help individuals to bolster their diminished personal control when facing scarcity. Nevertheless, we also observed that the subjects still distributed a considerable amount of resources to their partner in the insufficient and highly insufficient conditions across all the experiments (37–43%), indicating the robustness of sharing behavior under scarcity. In our opinion, resource sharing in these experiments was not driven by self-serving purposes, because: (i) our task design did not include a reciprocity mechanism for one’s partner to return the favor; (ii) according to the cover story, subjects’ decision would be confidential to their partner. That is to say, sharing resources with a partner could not increase either the possibility to be repaid in the future (direct reciprocity) or social reputation (indirect reciprocity) (Nowak 2006). In this regard, our behavioral data not only show that sharing behavior resists resource scarcity, but also indicate that this phenomenon could be driven by nonreciprocal altruism (Belk 2010).

Across three experiments, we have consistently found that the subjects with a higher level of empathy were more willing to unilaterally share limited resources to anonymous others, so as to alleviate the pain experience that those people may receive. As pointed out by many researchers, one of the other-oriented motives for altruistic behavior is to reduce the suffering of another person (Pavey et al. 2012; Batson et al. 2015); therefore, it is not surprising that empathy boosts altruistic sharing (Eisenberg and Miller 1987). Previous studies have indicated that affective and cognitive empathy show different relationships with sharing behavior (Decety and Yoder 2016; Oliver et al. 2016). Our results seem to highlight the importance of cognitive but not affective empathy, which may be partially caused by the paradigm itself. The cognitive aspect of empathy enables us to take other’s perspectives and make accurate predictions of other people’s needs (de Vignemont and Singer 2006). In the paradigm scenario, the negative consequences of resource scarcity for other people are yet to come; perspective-taking and socio-moral reasoning (associated with cognitive empathy) may therefore be more important for individuals to predict these consequences and take actions to avoid them, compared with the ability to experience others’ emotional states (associated with affective empathy). Follow-up studies may examine whether the effect of affective empathy would become stronger when subjects directly observe other people suffering from some consequences of scarcity (e.g. Singer et al. 2004; Bufalari et al. 2007).

In the field of neuroscience, it has been recently acknowledged that resource scarcity affects brain activation patterns (Huijsmans et al. 2019; Krosch and Amodio 2019) and even brain structure (Luby et al. 2013; Hair et al. 2015; Noble et al. 2015). This study detected that the rTPJ, mPFC, and PCC activations significantly decreased in the insufficient/highly insufficient conditions compared with the sufficient condition, all of which are key brain regions of a core mentalizing network that generalizes beyond experimental tasks and modalities (Van Overwalle 2009; Schurz et al. 2014). As we pointed out above, an altruistic decision to share resources with other people could be driven by the understanding of other people’s future states (e.g. suffering from malnutrition because of famine). This understanding might be inhibited under resource scarcity, since people focus more on their own benefits in a scarcity mindset (Holland et al. 2012; Roux et al. 2015). While the rTPJ and mPFC were consistently activated in all contrasts, the PCC activation was mainly driven by the difference between sufficiency and highly insufficiency. To understand these results, it should be noted that a main function of the PCC in mentalizing is mental imagery (i.e. to represent the perspective of another person) according to previous studies (Schurz et al. 2014). In our opinion, it is possible that the PCC is significantly activated only when the image of others’ feelings is highly arousing (e.g. in highly insufficient scenarios). Meanwhile, the main cognitive function of the rTPJ is to utilize all available information to predict others’ mental states, while the mPFC might be associated with the processing of socially or emotionally relevant information about others (Aichhorn et al. 2006; Saxe and Powell 2006) or integrating these information to compute “social value” (Decety et al. 2004; Behrens et al. 2008). Consequently, the participation of these regions is necessary for most experimental scenarios involving others.

The DCM analysis demonstrated an inhibition effect on the functional connection from the rTPJ to the mPFC when the resource was insufficient/highly insufficient. Considering the main functions of the rTPJ and mPFC in the literature (see above), this result indicate that resource sufficiency modulates the influence of other-oriented factors (e.g. other’s feelings and needs), and thus, these factors are weighted less important in the calculation of integrative value to guide decision-making (Zhang and Gläscher 2020). Furthermore, we also found that this inhibition effect was significantly correlated with the cognitive empathy traits, such that individuals with higher scores in cognitive empathy trait showed weaker inhibitions of insufficiency on the connection from the rTPJ to the mPFC. These results indicated a possibility that the functional connections within the cognitive empathy network were sensitive to resource sufficiency. By inhibiting the neural activities and connections between the key regions of this network (e.g. the rTPJ and mPFC), resource scarcity suppresses sharing behaviors and promotes a stronger self-serving bias. However, more empathic individuals, especially those with a higher level of cognitive empathy, might be less susceptible to resource scarcity, and thus, they would still share their resources to a comparable degree as they do when resources are sufficient.

Our final experiment reveals that oxytocin administration was effective to modulate resource sharing under scarcity, but only for those with a higher level of cognitive empathy. Specifically, the behavioral tendency of sharing resources with others was less likely to change between sufficiency and insufficiency (i.e. the insufficient/highly insufficient conditions) after the high-empathy group received intranasal oxytocin (compared with placebo), but there was no significant effect of oxytocin in the low-empathy group. Again, these results confirm the relevance of cognitive empathy to altruistic sharing behavior under scarcity. The relationship between oxytocin and cognitive empathy is supported by Quintana and Guastella (2020), which pointed out that oxytocin affects cognitive functions such as social learning and social adjustment. In our opinion, the current findings could help explain heterogeneous findings regarding oxytocin-treated subjects’ social behavioral patterns in the literature (Schiller et al. 2020), that is to say, the individual difference in empathy should be taken into account. Because of practical limitations, we did not investigate the neural underpinnings of the influence of oxytocin on resource sharing. We expect that oxytocin administration would enhance the strength of the functional connection from the rTPJ to the mPFC, especially for those having a high-empathy level.

Some “incidental findings” may also be worth noting, though they are not the main focus of this study. First, the behavioral results of our pilot experiment show that manipulating the threat level (4/8/12 s) did not directly affect the sharing rate or interact with the sufficiency factor, though the reaction time increased as a function of threat level. This finding suggests that despite the total amount of resource, people are more concerned about its scarcity status. Second, according to the behavioral and neuroimaging data from experiment 1 and 2, the differences between the insufficient and the highly insufficient condition did not reach statistical significance, indicating that these two conditions were essentially homogeneous. These results may reflect a binary evaluation system that generally distinguishes between resource sufficiency and insufficiency but is not sensitive to a specific level of insufficiency, possibly because scarcity has been shaped by evolution as an “alarm signal” in the human brain. Follow-up studies are awaited to test this hypothesis with alternative techniques (e.g. brain stimulation).

To sum up, this study reveals that although resource insufficiency significantly suppresses the behavioral tendency to altruistically share resources with unrelated individuals, this prosocial tendency still shows robustness under scarcity, which might be sustained by the mentalizing network in the brain. Furthermore, resource sharing and its neural mechanisms were sensitive to personal level of empathy, especially its cognitive component; oxytocin administration enhanced the tendency of sharing only among those subjects with a higher level of empathy. While it is reasonable to share resources with those who could return the favor (Goodman and Gareis 1993), this reciprocity mechanism does not always work in real life. Instead, many people are willing to sacrifice their own resources for the benefit of unrelated others when no further interaction is expected (Edele et al. 2013). Such sharing behaviors (e.g. anonymous donation), which are considered desirable from a collective perspective, are fundamental to establish and maintain common welfare in society (Moll et al. 2006). Regarding that, the current findings may help unravel the motivational basis of prosociality with behavioral and neurobiological evidence (see also Decety 2011), as well as different relations between empathy and prosocial behavior.

Finally, a few limitations and future directions should be addressed. First, alternative task design should be applied to examine the generalizability of the current findings, seeing that the noise administration in this study may not be comparable to the harmful consequence of resource scarcity (e.g. malnutrition or dehydration) in real life (see also Nosek et al. 2022). It should also be noted that social decision-making is generally more self-serving in the appetitive dimension than the aversive dimension according to previous studies (Crockett et al. 2014; Lockwood et al. 2017, 2020; Lengersdorff et al. 2020). Considering that, future studies should directly compare individual prosocial tendency in sharing decisions, as well as the influence of empathy, between these dimensions (see also Lockwood et al. 2016). The selection of stimulus modality (e.g. narrative vs. photographs or videos) might also be an issue, which significantly affects the involvement of cognitive/affective empathy in a given task scenario (Molenberghs et al. 2016). Last but not least, many studies have shown that both endogenous and intranasal oxytocin strengthen intergroup conflict (Zhang et al. 2019; Han et al. 2020); therefore, follow-up research should compare the impact of oxytocin on resource sharing between in-group and out-group conditions.

## Funding

This work was supported by the National Natural Science Foundation of China (nos. 32171013, 31871109, 31900779, 32071083, 32020103008, 31800944) and the Major Project of National Social Science Foundation (19ZDA363).

*Conflict of interest statement.* The authors declare no competing interests in relation to the subject of this study.

## Authors’ contributions

FC designed the study; JL and XH conducted the experiments and collected data; JL and XH analyzed data; FC, RG, YJ, JL, and YJL wrote the paper.

## Declaration of ethics

All procedures performed in this study were in accordance with the 1964 Helsinki declaration and its later amendments or comparable ethical standards. The local ethics committee approved the experimental protocol.

## Supplementary Material

Supplementary-1_bhac017
