# Supplementary material for: How resource sharing resists scarcity: the role of cognitive empathy and its neurobiological mechanisms
Source: Cereb Cortex. 2022 Feb 4;32(23):5330–42. doi: 10.1093/cercor/bhac017 (PMC9712734; doi:10.1093/cercor/bhac017)
Supplement: Supplementary-1_bhac017 [file supplementary-1_bhac017.docx]

How resource sharing resists scarcity: the role of cognitive empathy and its neurobiological mechanisms

***Supplementary***

#### *S1 Pilot experiment*

**Methods**

***Subjects***

Forty-three right-handed subjects were recruited from Shenzhen University to join in the pilot experiment. One of them was rejected due to failed recording, two of them were rejected due to their disbelief of the cover story, leaving thirty-nine subjects (20 women, age: 20.81 ± 1.3 years [mean ± s.d.]). Subjects were screened for a history of neurological disorders, brain injury, and developmental disabilities. All had a normal or corrected-to-normal vision. The study was conducted according to the ethical guidelines and principles of the Declaration of Helsinki and was approved by the Medical Ethical Committee of Shenzhen University Medical School. Informed consent was obtained from all subjects after they fully understood the procedures.

***Experimental Procedures***

**Please see Experiment 1: Experimental Design in the main text for details of the procedures. The experiment applied a two-factor within-subject design. The first factor was noise duration (*threat level*) for each subject (high: 12 s; medium: 8 s; low: 4 s). The second factor was the *sufficiency* of the relieving resource (sufficient: two times as long as the noise duration; insufficient: the same length with the noise duration; highly insufficient: only half of the noise duration). Please note that "sufficiency" here was defined according to whether the relieving resources were enough for both players. For example, if the real subject received 8 s of noise in a given trial, then the resource amount was 2 × 8 = 16 s in the "sufficient" condition, 8 s in the "insufficient" condition, and 4 s in the "**highly **insufficient" condition. Nine conditions (3 × 3) were generated accordingly. In each trial, the real subject was shown (1 s) how many seconds of noise s/he and the confederate might hear. After a 0.5 s fixation, the subject was shown (1.5 s) how many seconds of "relieving resource" s/he might receive. Then the subject decided "how many resources s/he want to keep for her/himself" by inputting the number of seconds with numerical keys on a keyboard placed in front of her/him. S/he had a maximum of 4 s to make this decision. After that, the subject observed the feedback (1.5 s) of her/his decision (i.e., how many seconds of noise administration s/he might be given) (Fig.** S1**a). The experiment consisted of 45 trials (5 trials per condition) lasting approximately 8 min.**

**After the experiment, subjects received remuneration and a debriefing was given by the experimenter. Additionally, to access whether individual empathic traits play a role in solving this resource dilemma, all subjects were asked to fulfill the Questionnaire of Cognitive and Affective Empathy (QCAE) (Reniers et al., 2011) during the recruiting process. They were required to fulfill a feedback questionnaire after the task and before debriefing, which included questions asking them whether they believed that their decision would influence the other person (Yes or No).**

**Results**

**Our feedback question showed that subjects believed in the cover story, that is, there was a real person playing as their partner during the task and their decision would influence both of them (40 out of 42 subjects answered YES on the feedback question). We run one-sample *t*-tests to compare the sharing rate under each condition with the random level (i.e., 0.5). Results showed that across different threat levels (4 s/8 s/12 s), the sharing rate was not significantly different from 0.5 under the sufficient condition (*ps* > 0.10). However, the sharing rate was significantly smaller than 0.5 under the insufficient and highly insufficient conditions (*ps* < 0.02), indicating that subjects were more likely to keep the resource for themselves in these conditions.**

**To examine the effects of *resource sufficiency* and *threat level* (i.e. noise duration), 3 (sufficiency: sufficient, insufficient, highly insufficient) × 3 (threat level: 4 s, 8 s, 12 s) repeated measures ANOVAs were conducted on the sharing rate and reaction time. Regarding the sharing rate, the main effect of resource sufficiency was significant** (*F*(2,78) = 11.21, *p* < 0.001, *η_p_^2^* = 0.22); the sharing rate was lower when resource was insufficient or highly insufficient (sufficient: 0.50 ± 0.01; insufficient: 0.43 ± 0.03; highly insufficient: 0.39 ± 0.03; sufficient vs insufficient: *p* = 0.001; sufficient vs highly insufficient: *p* < 0.001; insufficient vs highly insufficient: *p* = 0.08). The main effect of threat level (*F*(2,78) = 0.37, *p* = 0.69, *η_p_^2^* = 0.01) and its interaction with resource sufficiency (*F*(4,156) = 0.12, *p* = 0.97, *η_p_^2^* = 0.00) were not significant (Fig. S1b).

Regarding the reaction time, **the main effect of** threat level **was significant** (*F*(2,78) = 45.35, *p* < 0.001, *η_p_^2^* = 0.54); subjects took a longer time to make decisions as the threat level increased (high [12 s]: 1556.88 ± 91.33 ms; medium [8 s]: 1332.71 ± 80.37 ms; low [4 s]: 1199.11 ± 67.12 ms; high vs medium: *p* < 0.001; high vs low: *p* < 0.001; medium vs low: *p* < 0.001). The main effect of **resource sufficiency** (*F*(2, 78) = 0.97, *p* = 0.39, *η_p_^2^* = 0.02) and the interaction between two factors (*F*(4,156) = 2.05, *p* = 0.09, *η_p_^2^* = 0.05) were not significant (Fig. S1c).

**We also run one-sample *t*-tests between the changing rate under each threat level and zero. Results showed that for all the three threat levels, the changing rate was significantly larger than zero (12 s: 0.20 ± 0.06; 8 s: 0.20 ± 0.06; 4 s: 0.19 ± 0.07; *ps* < 0.007). One-way ANOVA showed that the changing rate between different threat levels was not significantly different (*p* = 0.85;** Fig. S1d**).**

**Pearson correlation analysis showed that the changing rate under all three threat levels was negatively correlated with the cognitive empathy score (high: *r* = -0.52, *p* = 0.001; medium: *r* = -0.44, *p* = 0.005; low: *r* = -0.40, *p* = 0.01) but not with affective empathy score (*ps* > 0.12)** (Fig. S1e)**.**


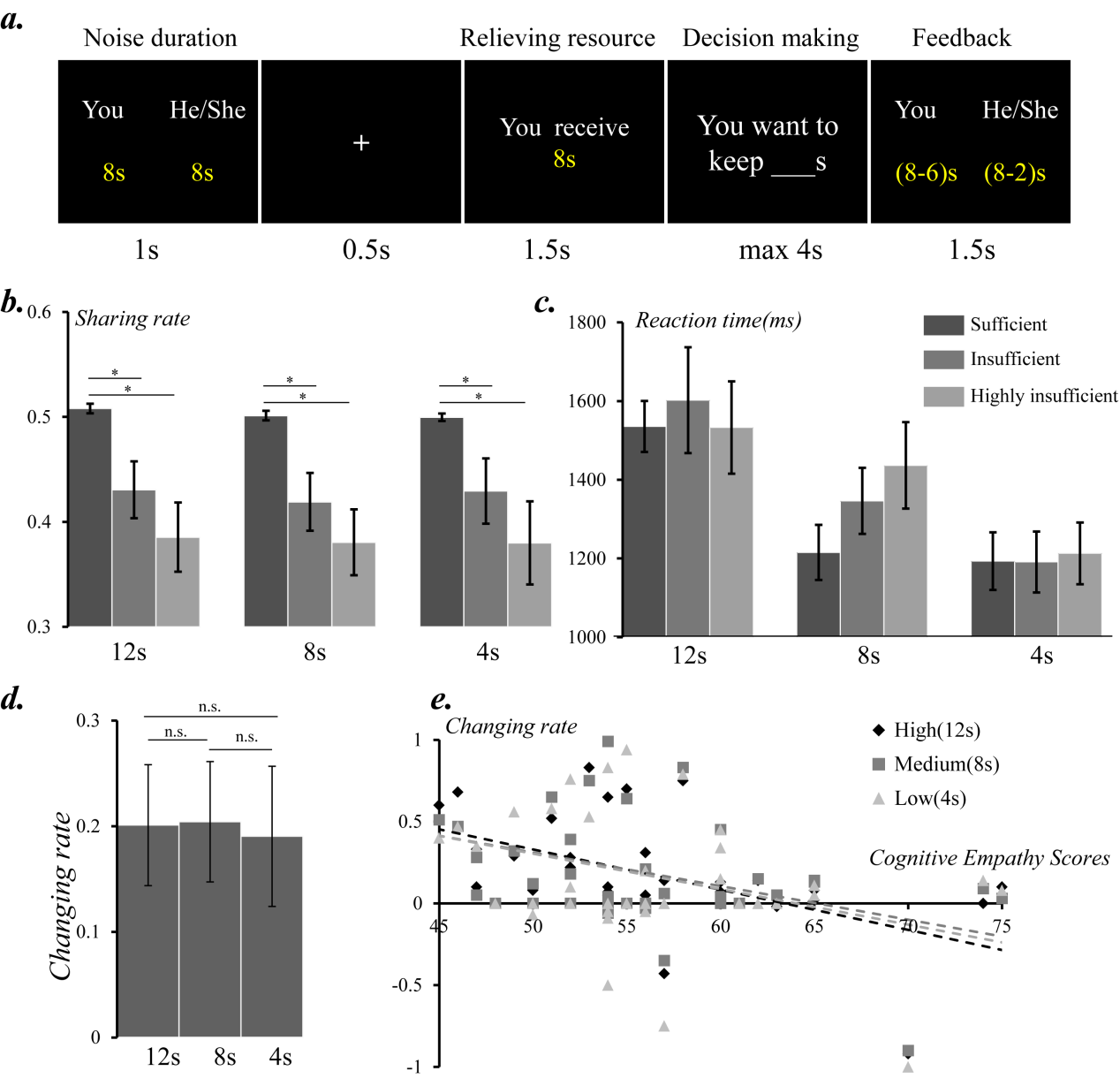


**Figure S1. Experimental design and results of the pilot experiment.** *a*. Structure of an example trial; *b*. Results of the sharing rate for each level of resource sufficiency; *c.* Results of the reaction time for each level of resource sufficiency; *d*. Results of the changing rate for each threat level; *e*. Correlations between the changing rate and cognitive empathy score for each threat level. n.s.: not significant.

#### *S2 The contrasts between either two of the three conditions*

We compared brain activation between either two of the three conditions (i.e., sufficient, insufficient, and highly insufficient) in Experiment 1. At the first-level analysis, the effect of experimental manipulation on regional blood oxygenation level-dependent responses was estimated with the general linear model using the three levels of relieving resource (sufficient, insufficient, and highly insufficient) as independent regressors. We then defined contrasts as: sufficient minus insufficient, sufficient minus highly insufficient, as well as insufficient minus highly insufficient.

In order to determine whether there was a similar pattern between “sufficient minus insufficient” and “sufficient minus highly insufficient,” a more liberal significance level as *p* < 0.001 uncorrected was used. The contrast of *sufficient > insufficien*t showed activation in bilateral temporoparietal junction (TPJ) (Montreal Neurological Institute [MNI] coordinate [-51, -75, 30], MNI coordinate [57, -66, 27]), medial prefrontal cortex (mPFC) MNI coordinate [9, 48, 45]). The contrast of *sufficient > highly insufficien*t revealed significant differences in the middle occipital gyrus (MNI coordinate [45, -69, 30]), TPJ (MNI coordinate [-48,-75, 30]), posterior cingulate gyrus (MNI coordinate [-6, -45, 33]), medial orbital part of superior frontal gyrus (MNI coordinate [6,54, -12]), middle temporal gyrus (MNI coordinate [-63, -12,-12]), as well as the medial and dorsal lateral part of the superior frontal gyrus (MNI coordinate [-21, 36, 45]), which were similar to the contrast of “sufficient minus insufficient” reported above (See Fig S2). Regarding the contrast of *insufficien*t *> highly insufficien*t, no significant difference was observed under *p* < 0.005 uncorrected.


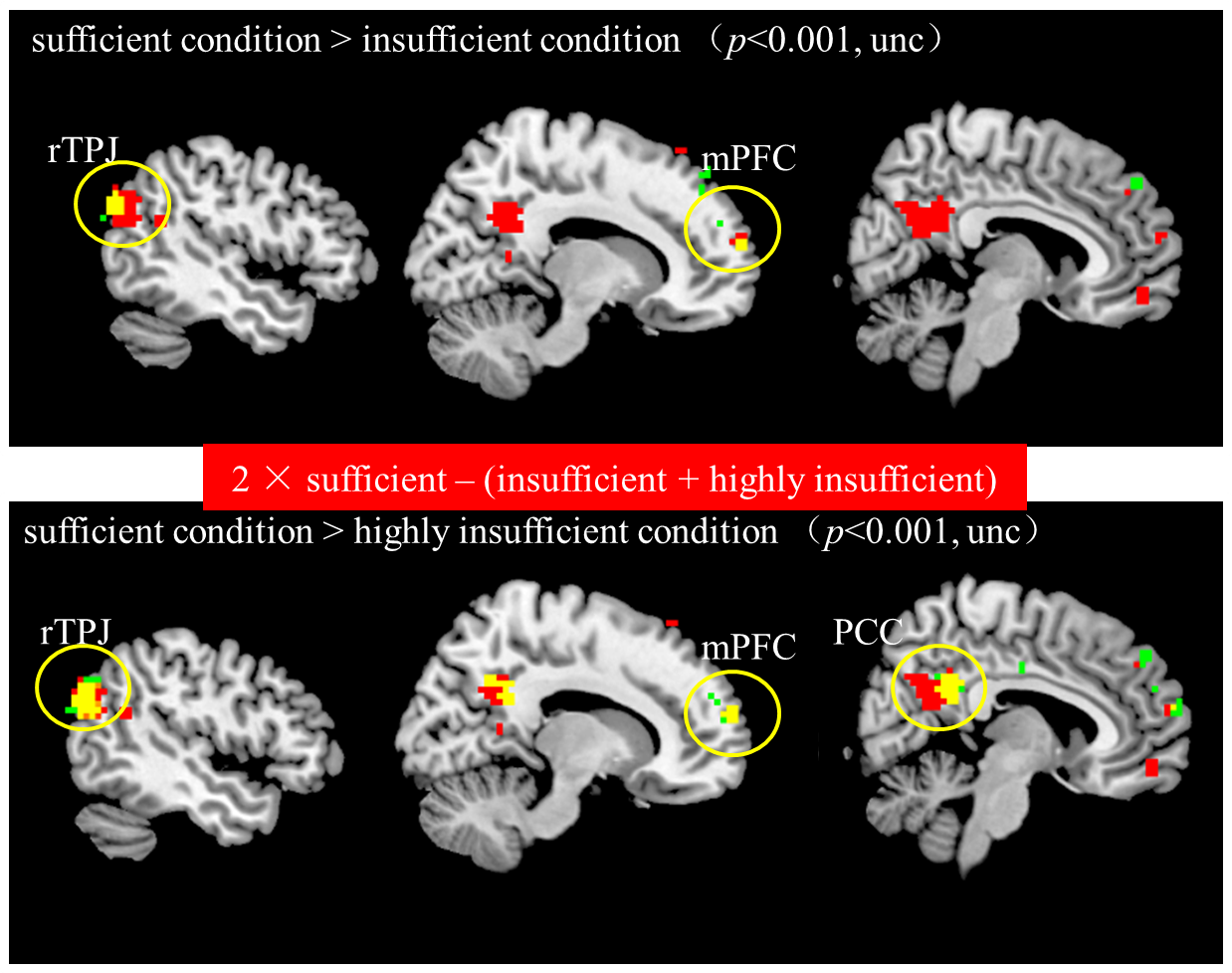


**Figure S2.** Whole-brain activations based on the contrasts of (sufficient condition > insufficient condition) (panel a, green), (sufficient condition > highly insufficient condition) (panel b, green), and their overlap with the contrast of (2 × sufficient condition – [insufficient condition + highly insufficient condition]) (red in panel a & b).

#### *S3 Dynamic causal modeling (DCM) and Bayesian Model Selection (BMS) results*


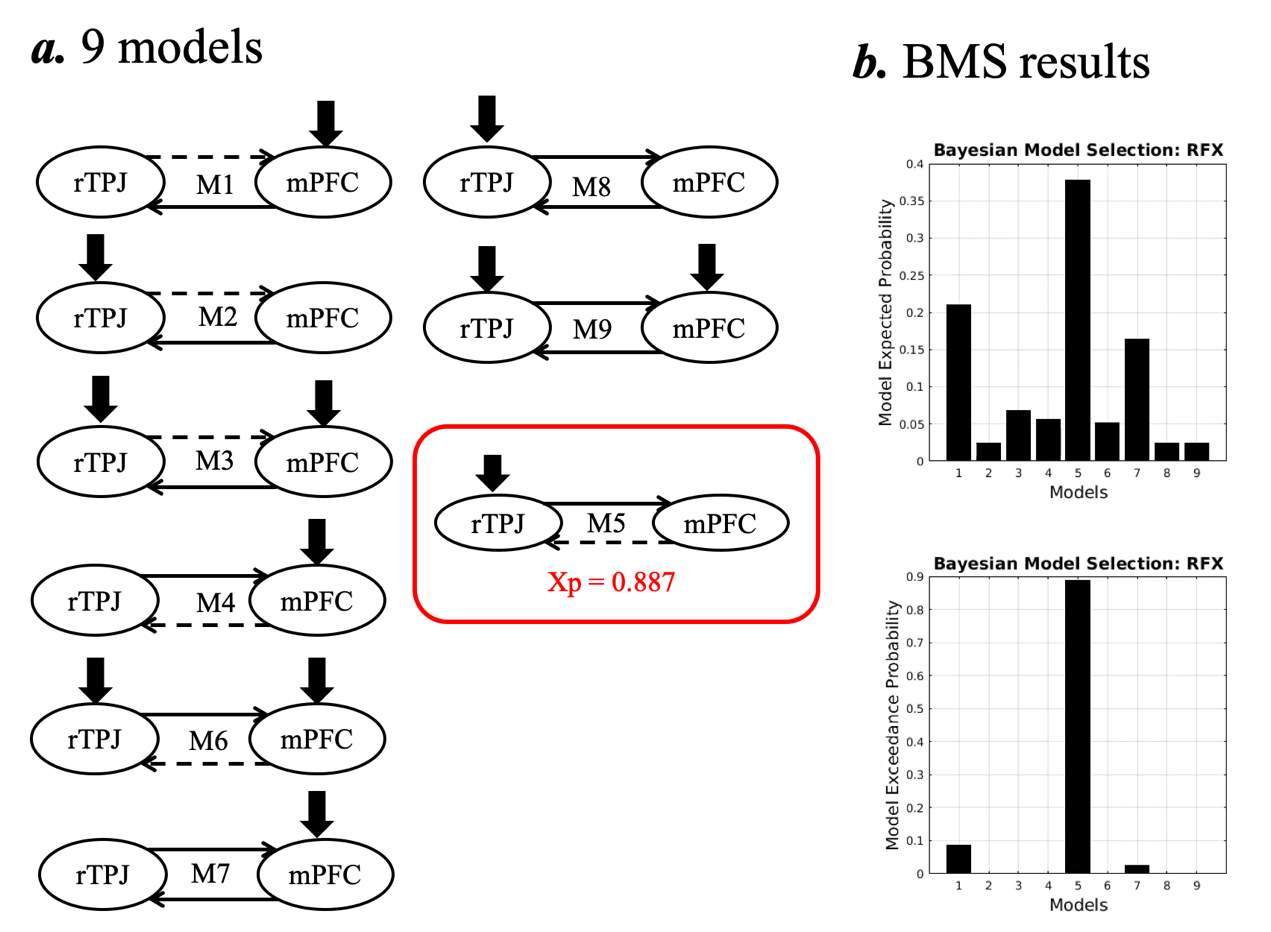


**Figure 3.** *a.* 9 models of the DCM: solid lines indicate the connections on which the modulatory effect worked; dashed lines indicate the instinct connections; black arrows pointing to the regions of interest indicate the input to the system; *b.* BMS results of the DCM.

**References**

Hu, J., Li, Y., Yin, Y., Blue, P. R., Yu, H., & Zhou, X. (2017). How do self-interest and other-need interact in the brain to determine altruistic behavior? *Neuroimage*, *157*, 598-611. <https://doi.org/10.1016/j.neuroimage.2017.06.040>

Knutson, B., Rick, S., Wirnmer, G. E., Prelec, D., & Loewenstein, G. (2007). Neural predictors of purchases. *Neuron*, *53*(1), 147-156. <https://doi.org/10.1016/j.neuron.2006.11.010>

Reniers, R. L., Corcoran, R., Drake, R., Shryane, N. M., & Völlm, B. A. (2011). The QCAE: A questionnaire of cognitive and affective empathy. *Journal of Personality Assessment*, *93*(1), 84-95. <https://doi.org/10.1080/00223891.2010.528484>
